# Revealing the Action Mechanism of WISP2 in Miscarriage Based Multi‐Omic Analysis and Experimental Validation

**DOI:** 10.1111/cbdd.70423

**Published:** 2026-11-05

**Authors:** Zhe Leng, Boyu Yan, Fang Wang, Xinyi Bian, Yu Huang, Hongjuan Lv, Zhou Jiang, Xianli Sun

**Affiliations:** ^1^ Department of Gynecology and Obstetrics, Women and Children's Hospital Qingdao University Qingdao Shandong China; ^2^ Department of Gynecology and Obstetrics Qingdao Shibei District Hailun Road Community Health Service Center Qingdao Shandong China; ^3^ Department of Otolaryngology, Women and Children's Hospital Qingdao University Qingdao Shandong China; ^4^ Department of Reproductive Medicine, Women and Children's Hospital Qingdao University Qingdao Shandong China; ^5^ Shandong Boaoke Biotechnology Co. Ltd Liaocheng Shandong China

**Keywords:** early pregnancy, E‐cadherin, recurrent miscarriage, WISP2

## Abstract

Recurrent miscarriage (RM) is a major unresolved issue in reproductive medicine. Although WISP2 is known to participate in multiple cellular processes, its function in trophoblast biology and pregnancy maintenance remains unknown. We performed single‐cell RNA sequencing on chorionic villus tissues from RM patients and normal controls to identify cell‐type‐specific transcriptional alterations, which led to the identification of WISP2 as a candidate gene in the SCT/EVT subpopulation. Fetal membrane samples (chorion) were obtained from Qingdao Women and Children's Hospital's Surgical Outpatient Department: 20 RM patients and 20 healthy controls. Using qRT‐PCR, Western blot, and immunohistochemistry to detect gene and protein expression. HTR‐8 cells were used to construct WISP2 overexpressing cells, and MTT, wound healing and Transwell experiments were performed for functional experiments. Eleven cell classes in the sample were demonstrated by single‐cell analysis. Among these cells were DSCs and SCT/extravillous trophoblasts (EVT). The level of WISP2 is low in SCT/EVT cells from RM patients. In chorionic villus tissues, both WISP2 and E‐cadherin were significantly upregulated in the RM group relative to the control group (all *p* < 0.05). Immunohistochemistry confirmed elevated WISP2 and E‐cadherin protein levels in RM chorionic villi (both *p* < 0.05), with predominant cytoplasmic and membranous localization, respectively. In HTR‐8 cells, WISP2 overexpression suppressed proliferation (35%, *p* < 0.01), migration (45%, *p* < 0.01), and invasion (50%, *p* < 0.001), upregulated E‐cadherin (*p* < 0.05), downregulated Vimentin (*p* < 0.05), and increased p‐ERK (*p* < 0.05) without altering total ERK1/2 (*p* > 0.05), suggesting that WISP2 promotes an epithelial‐like phenotype accompanied by ERK pathway activation. In summary, WISP2 is dysregulated in RM and suppresses trophoblast invasion and EMT in vitro, suggesting a potential role in RM pathogenesis, though in vivo validation is required.

## Graphical abstract

In this study, the single‐cell atlas at the maternal–fetal interface in normal and recurrent miscarriage (RM) samples showed 11 cell types, including DSCs and SCT/EVT cells. In SCT/EVT cells, WISP2 expression was markedly decreased in the RM cases. Elevated levels of WISP2 and E‐cadherin were detected in villous tissues of patients with RM. The experimental overexpression of WISP2 in HTR‐8 cell lines markedly suppressed cellular proliferation, migratory capacity, and invasive potential.

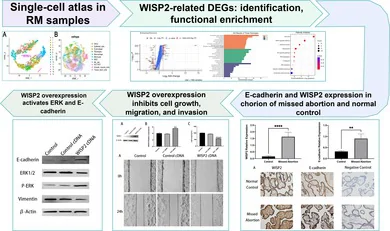

## 1 Introduction

In the early stages of pregnancy, the embryo or fetus may stop developing, and ultrasound examination reveals an empty sac or no fetal heartbeat (Marwah et al. 2016). This is common in women of childbearing age. Moreover, pregnancy loss during early pregnancy accounts for approximately 31% of all confirmed pregnancies (Zinaman et al. 1996). Missed abortion brings considerable physical and financial burdens to the mother. It also brings a serious psychological burden to the mother and even the whole family and even affects the subsequent pregnancy. Additionally, women who have experienced missed abortions, especially those with recurrent spontaneous abortions, often show fear and anxiety and are even on the verge of despair (Volgsten et al. 2018). So far, a variety of factors leading to missed abortions have been identified, including genetic factors, endocrine disorders, uterine abnormalities, immune disorders, endometriosis, prethrombotic state, infection, malnutrition, environmental factors, psychological factors, and infection (Bulletti et al. 1996; Clifford et al. 1994; Hatasaka 1994). Consequently, comprehensive elucidation of the underlying biological pathways associated with pregnancy loss would facilitate improved clinical interventions for affected individuals.

Recurrent miscarriage (RM), also referred to as recurrent pregnancy loss (RPL), is defined as two or more pregnancy losses before 20–24 weeks of gestation and represents a clinically significant reproductive disorder affecting approximately 1%–2% of women of reproductive age (Bender Atik et al. 2018; Dimitriadis et al. 2020). Unlike sporadic abortion (usually attributed to random chromosomal abnormalities), RM is more likely to reflect potential maternal pathological conditions, including immune dysregulation, decidualization defects, and impaired trophoblast invasion (Brosens et al. 2022; Cao et al. 2022; Uța et al. 2024). At present, up to 40%–60% of RM cases are classified as unexplained (Dimitriadis et al. 2020; Papas and Kutteh 2020), and its pathogenesis is still not fully understood.

WISP2, also designated as CCN5, is a secreted matricellular protein belonging to the CCN family and serves as a downstream signaling molecule of the Wnt/β‐catenin pathway (Liu et al. 2017). Unlike typical CCN members containing four functional domains (Insulin‐like growth factor binding proteins, von Willebrand factor C, thrombospondin‐1, and cysteine‐rich domain), WISP2/CCN5 uniquely lacks the C‐terminal CT domain, which may give it unique, context‐dependent functional properties (Brigstock 2003; Leask and Abraham 2006; Sabbah et al. 2011). Successful pregnancy depends largely on the precise regulation of trophoblast cell invasion, spiral artery remodeling, and the formation of a tolerant maternal‐fetal immune microenvironment (Balasundaram and Farhana 2026; Soares et al. 2014). Emerging evidence suggests that WISP2 may be functionally related to these processes. Shi et al. found that WISP2 can promote the survival of decidual stromal cells (DSCs) and weaken the cytotoxicity of decidual natural killer (dNK) cells through the IGF1 signaling pathway at the maternal‐fetal interface (Shi et al. 2021), indicating that it has a potential role in regulating the decidual immune microenvironment. Their study also found that estrogen and progesterone up‐regulated the expression of WISP2 in DSCs. These two steroid hormones are essential for maintaining pregnancy, suggesting that WISP2 may be a downstream mediator of hormone regulation in early pregnancy.

WISP2 has a bidirectional regulatory effect on cell proliferation and its effects are jointly influenced by the type of cell and the surrounding environment. For example, in hormone‐responsive non‐invasive breast tumors, elevated synthesis of epidermal growth factors or insulin‐like growth factor‐I is a crucial prerequisite for the mitogenic properties of these molecules. Overexpression of these signaling proteins creates a permissive environment that facilitates cellular proliferation in ER‐positive malignancies (Banerjee et al. 2003; Dhar et al. 2007; Sengupta et al. 2006). The gene demonstrates selective growth suppression activity within vascular smooth muscle cells and prostate carcinoma cell lines (Lake et al. 2003). Nevertheless, the direct involvement of WISP2 in trophoblast biology, especially its effects on trophoblast proliferation, migration and invasion, has not been systematically studied. In addition, whether abnormal WISP2 expression leads to the pathogenesis of RM by destroying trophoblast function is still completely unknown.

Given that insufficient trophoblast invasion is a recognized marker of RM (Pei et al. 2026), and WISP2 has been shown to regulate cell migration and invasion in other biological contexts (Fritah et al. 2008; Ji et al. 2014), we hypothesized that WISP2 may play a key role in regulating trophoblast invasion, and its dysregulation may be related to abortion. To investigate WISP2's involvement in pregnancy loss, we performed a comparative analysis of WISP2 and E‐cadherin expression patterns in healthy pregnancy and first‐trimester miscarriage samples. Using an engineered WISP2‐overexpressing HTR‐8 trophoblast cell line, we systematically examined WISP2's functional impact on critical cellular behaviors including proliferation capacity, migratory potential, and invasive properties. Furthermore, our study revealed the molecular pathways through which WISP2 contributes to spontaneous abortion pathogenesis. This study provides direct evidence supporting the role of WISP2 in miscarriages.

## 2 Materials and Methods

### 2.1 Source of Data Set

In this research, single‐cell class data were used, which were derived from the GSE214607 dataset. The data set information was as follows: five samples of normal women and three samples of RM patients. Samples were decidua and villi from females. In addition, the relevant data of normal samples and RM samples in GSE161969, GSE111974, and GSE165004 datasets are also downloaded. The samples were obtained from public databases and therefore did not require ethical approval from local institutions.

### 2.2 Data Analysis Process

For the dataset to be analyzed, quality control was performed on the data using “Seurat” (exclusion criteria: Genes < 250 or > 5000, mitochondrial contamination > 25%). The top 3000 highly variable genes were analyzed. Further, principal component analysis and extraction of principal components were conducted. Subsequently, the extracted principal components were used to perform cell annotation through “SingleR.” Syncytiotrophoblast/Extravillous Trophoblast (SCT/VET) was annotated in combination with the literature (15). Then, the “Seurat” software was used to conduct expression level difference analysis for the target genes WISP2 to obtain differentially expressed WISP2‐related gene, with thresholds of |log2 fold change (log2FC)| ≥ 1 and adjusted *p* value < 0.05. For the core gene WISP2, gene ontology (GO) and Kyoto Encyclopedia of Genes and Genomes (KEGG) enrichment analyses of these differentially expressed genes were performed using the clusterProfiler package (version 4.6.0). A nominal *p* value < 0.05 was used as the initial screening threshold, and the Benjamini–Hochberg method was applied for multiple‐testing correction; functional annotations and pathway terms with an adjusted *p* value (false discovery rate, FDR) < 0.05 were considered statistically significant.

### 2.3 Patients and Tissue Collection

Researchers gathered 20 instances of RM along with 20 healthy control subjects at the Qingdao Women and Children's Hospital. The RM patients were defined as those with two or more consecutive spontaneous pregnancy losses before 20–24 weeks of gestation, all occurring in the first trimester (6–10 weeks) of the index pregnancy. The healthy control group consisted of 20 age‐matched women with normal first‐trimester pregnancies (6–10 weeks of gestation) who underwent elective termination of pregnancy for non‐medical reasons at the same hospital during the same period. Controls had no history of spontaneous abortion, preterm birth, or adverse pregnancy outcomes, and all had at least one successful full‐term pregnancy prior to enrollment. The exclusion criteria were chromosomal irregularities before or during gestation, autoimmune disorders, structural abnormalities in the reproductive system, infections of the reproductive tract, chronic conditions, genetic disorders, and other systemic illnesses. Individuals exposed to toxic substances or undergoing hormonal therapy were excluded from this study. Pregnancy duration was verified between Weeks 6 and 10 using the last menstrual period and ultrasound assessments.

Chorionic villus samples were obtained from all participants under ultrasound‐guided vacuum aspiration or dilation and curettage (D&C) during surgical procedures. Immediately after collection, the villous tissues were carefully separated from decidual tissue and blood clots under a stereomicroscope and washed three times with ice‐cold phosphate‐buffered saline to remove maternal blood contamination. A total of 20 samples were collected from the RM group and 20 from the healthy control group. Each specimen was divided into two portions: one was preserved at −80°C for protein or RNA isolation, while the other was immersed in 4% formaldehyde for subsequent immunohistochemical analysis. Patient‐specific data are presented in Table S1. Written informed consent was obtained from all participants, and ethical approval was granted by the Institutional Review Board of Qingdao Women and Children's Hospital (Ethics Approval Number: QFELL‐YJ‐2020‐58).

### 2.4 Quantitative Real‐Time PCR (qRT‐PCR)

Total RNA was isolated from chorionic villus samples across all experimental groups using the MolPure RNA extraction system, followed by reverse transcription for conversion to cDNA. In the quantitative real‐time PCR process, the Hieff qPCR SYBR Green Master Mix kit (brand: Yeason, origin: China) and the QuantStudio3 system (brand: Samufei, origin: USA) were used. Relative mRNA expression levels were calculated using the 2^−ΔΔCt^ method, with GAPDH serving as the internal reference gene. Specific primers were designed for the WISP2 gene, with the forward sequence AGGTATGGCAGAGGTGCAAG (5′–3′) and the reverse sequence AGGGGAAGTCGGAGAGAAAG (5′–3′). The specific primer sequences for the E‐cadherin gene are ATTTTTCCCTCGACACCCGAT (5′–3′) and TCCCAGGCGTAGACCAAGA (5′–3′). For transcript quantification, glyceraldehyde‐3‐phosphate dehydrogenase (GAPDH) served as the endogenous control, utilizing the forward primer sequence ACAACTTTGGTATCGTGGAAGG (5′–3′) and reverse primer GCCATCACGCCACAGTTTC (5′–3′).

### 2.5 Western Blot

The chorion was washed twice with cold PBS (pH 7.4). High‐speed RIPA buffer (pH 7.0) from Epizyme, China, was used to lyse the tissue. First, 200 g of the tissue lysed in 15 mL of high‐speed RIPA buffer was spiked with 1% protease inhibitors and phosphatase inhibitors (Epizyme) for 2 h at 4°C, and centrifuged at 16,000×*g* for 20 min at 4°C. A clear supernatant was obtained, and the sample buffer was added. The samples were boiled for 10 min in water, and the protein content in the supernatant was calculated using a BCA assay kit from Epizyme (China). The same concentration of the protein after the same centrifugation was washed five times with phosphate‐buffered saline PBS. Finally, an ECL ultrasensitive luminescence reagent kit (Epizyme, China) was used to detect the membrane. The following primary antibodies were used for Western blotting: anti WISP2 (1:1000), anti‐E‐cadherin (1:1000), anti‐ERK1/2 (1:10,000), anti‐p‐ERK (1:1000), anti‐vimentin (1:1000), and anti‐β‐actin (1:5000, Abcam, UK) as the internal loading control.

### 2.6 Immunohistochemistry

Immunohistochemical techniques were used for the localization and expression analysis of WISP2 and E‐CAD in the villi. Villus samples were fixed in 4% paraformaldehyde overnight at 4°C, embedded in paraffin, and sliced into 5 μm thick sections. The samples were deparaffinized using xylene. Subsequently, the samples were rehydrated using ethanol at a concentration gradient (100%–70%). The sample slices were soaked in a citric acid buffer solution with a concentration of 0.01 mol; the immunohistochemical experiment was performed after the tissue was put into microwave oven to be antigen retrieval according to the guidance of SP reagent kit (ZSGB‐BIO) of our country. The DAB staining kit (ZSGB‐BIO, Chinese) was used for staining diaminobenzidine, and positive outcomes were showed by brown staining. As mentioned, an Olympus DP80 microscope with its DP controlling software was used for collecting HD slice images simultaneously.

### 2.7 Cell Culture and Transfection

The HTR‐8 human chorionic trophoblast cell line was purchased from Shanghai Zhong Qiao Xin Zhou Biotechnology Co. LTD. RPMI 1640 medium (containing 10% fetal bovine serum, from Israel Biological Industries) was used to culture the cells. A 0.25% trypsin solution (provided by Beirui, China) is used for cell subculture, seeding cells into 6‐well and 96‐well cell culture plates. WISP2 and control plasmid are transfected with JetOPTIMUS reagent (from Polyplus, USA). All the WISP2 plasmids were purchased from Youmei Biotechnology (Hunan, China).

### 2.8 MTT Analysis

Cells were seeded in a 96‐well plate at 5 × 10^3^ cells/well and transfected with WISP2 plasmid for 10 h. After 72 h of incubation, 20 μL of MTT (5 mg/mL in phosphate‐buffered saline, Sigma‐Aldrich, USA) was added to each well for 4 h. The formazan crystals were dissolved in 150 μL DMSO, and the absorbance was measured at 490 nm using a microplate reader.

### 2.9 Wound Healing Analysis

Cells were transfected with the WISP2 plasmid for 10 h. Use the pipette tip to make a cut at the middle position of the cell culture. The detached cells remaining at the incision site were washed with PBS. Next, RPMI 1640 culture medium containing 2% fetal bovine serum was added to each well, and the cells were maintained at 37°C in a 5% CO_2_ environment. The scratches were observed and photographed under a microscope at 0, 24, and 48 h, respectively, and their changes were recorded and statistically analyzed.

### 2.10 Transwell Invasion Assay

Transwell cell culture chambers (Corning, US) were used to assay the invasion ability of transfected HTR‐8 cells. To determine invasiveness, the chambers were coated with a Matrigel admixture (BD, USA). The cell samples under cultivation were transplanted into the upper container, which was filled with serum‐free RPMI 1640 nutrient solution, with a cell count of approximately (5 × 10^4^ cells). Cells, grown at 37°C and 5% CO_2_ for 24 h on the lower chamber with RPMI 1640 nutrient solution with 10% of fetal bovine serum, are washed, fixed, and then stained by hematoxylin for 20 min; the cells that migrate through the lower barrier Transwell are counted and captured on a microscope.

### 2.11 Statistical Analysis

All statistical analyses were performed using GraphPad Prism 9. Data are presented as the mean ± standard error of the mean (SEM). For comparisons between two independent groups, statistical significance was determined using two‐tailed unpaired Student's *t*‐test. For comparisons involving more than two groups, one‐way analysis of variance (ANOVA) was applied, followed by Tukey's multiple comparisons test for post hoc pairwise comparisons. A *p* value < 0.05 was considered statistically significant.

## 3 Result

### 3.1 Single‐Cell Analysis of Normal and RM Patient Samples

After conducting data screening on the single‐cell data of normal and patient samples in the dataset, we obtained 112,528 cells that met the criteria for further analysis. Among them, the normal patient's decidual source cells are 36,219, and the RM decidual source cells are 25,582. Normal villus cells are 25,303; RM villus cells are 25,424. Unsupervised clustering and visualization using t‐SNE revealed distinct cellular populations (Figure 1A). Further analysis using SingleR confirmed the existence of 11 cell types. Among these 11 types of cells, there are DSCs and SCT/EVT cells, both of which are known to play critical roles at the maternal–fetal interface (Figure 1B).

**FIGURE 1:**
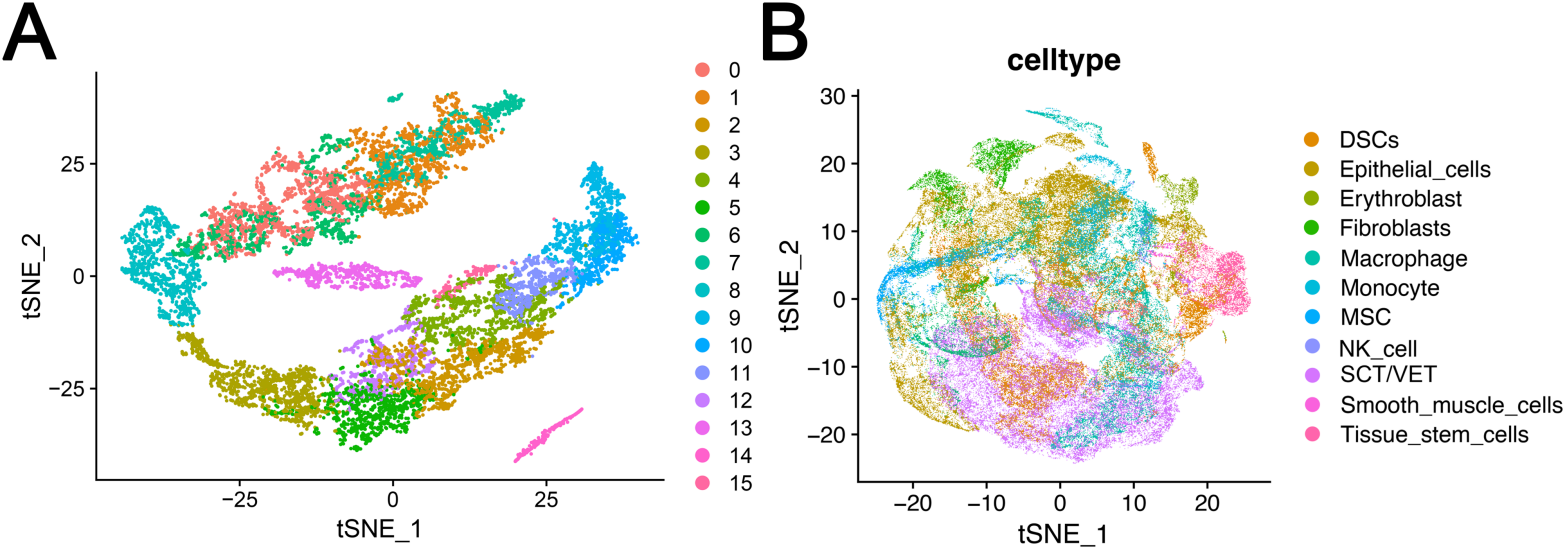
Single‐cell analysis of normal and RM patient samples. (A) Cluster analysis of cell samples. (B) Cell annotations for these clusters.

### 3.2 Functional Exploration of the Core Gene WISP2

Firstly, the expression of WISP2 in different cell populations was analyzed. Results revealed that WISP2 was predominantly expressed in DSCs, whereas relatively lower expression was observed in SCT/EVT cells (Figure 2A). Subsequently, given the prominent expression of WISP2 in DSCs and its previously reported role in regulating decidual immune homeostasis, cells were subsequently stratified into WISP2‐high and WISP2‐low groups to explore WISP2‐associated transcriptional programs at the maternal‐fetal interface (Figure 2B). Differential expression analysis of these two groups yielded 1372 differentially expressed WISP2‐related genes (Figure 2C). Through enrichment analysis of these genes, it was explained that they were mainly concentrated in ribonucleoprotein complex biogenesis, cellular components in chromosomal regions, and molecular functions related to cadherin binding (Figure 2D–F). On the pathway, the regulation of these gene cell cycle, MAPK signaling pathway, and adherens junctions‐related pathways (Figure 2G). We further compared the copy number of WISP2 in control and RM patients in three datasets (GSE161969, GSE111974, and GSE165004). The results showed that WISP2 was significantly highly expressed in RM patients in these three datasets (Figure S1A–C).

**FIGURE 2:**
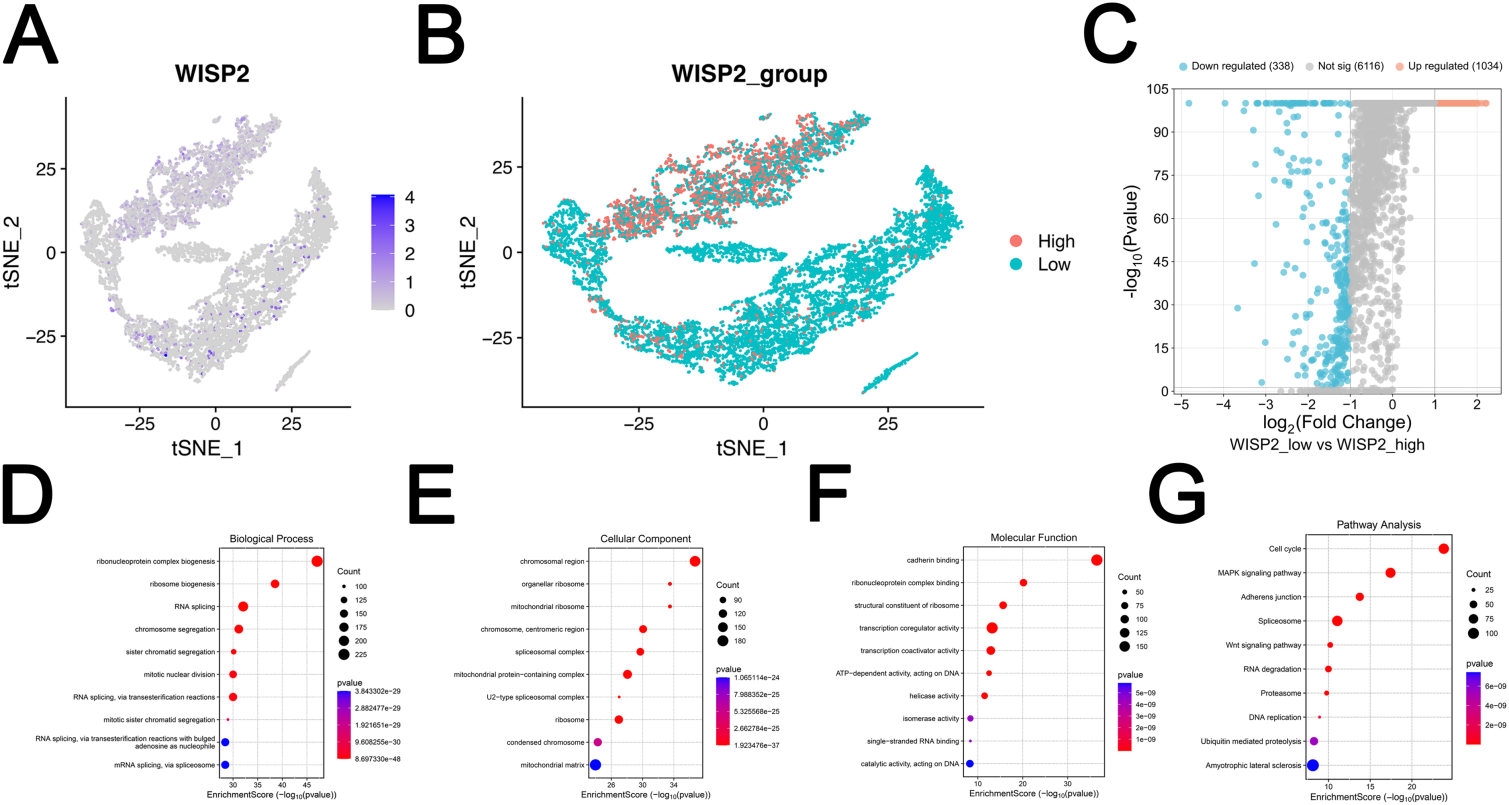
WISP2 functional exploration of the core gene WISP2. (A) Analysis of tSNE specific to WISP2. (B) Distribution of high and low expression of WISP2 cells. (C) Differential expression analysis was performed based on the high and low expression of WISP2. (D–F) GO enrichment analysis of BP (D), CC (E), and MF (F). (G) KEGG enrichment analysis.

### 3.3 E‐Cadherin and WISP2 mRNA Expression in the Chorion of Recurring Miscarriages and Normal Control Detected by Quantitative Real‐Time PCR

This study showed that the villi of RM exhibited high WISP2m RNA expression when compared to the normal control, as measured by the quantitative real‐time PCR (qRT‐PCR) method (*p* < 0.0001) (Figure 3A). The invasion of EVT requires a partial EMT process, and E‐cadherin is a typical epithelial marker, and its up‐regulation is associated with decreased migration and invasion (Luan et al. 2024). Therefore, we also determined E‐cadherin. Therefore, we also determined E‐cadherin. The mRNA level of E‐cadherin in the chorion of RM was significantly higher than that in the chorion of normal controls (*p* < 0.01) (Figure 3B). Substantial upregulation of WISP2 and E‐cadherin genes has been confirmed in the chorion in miscarriage.

**FIGURE 3:**
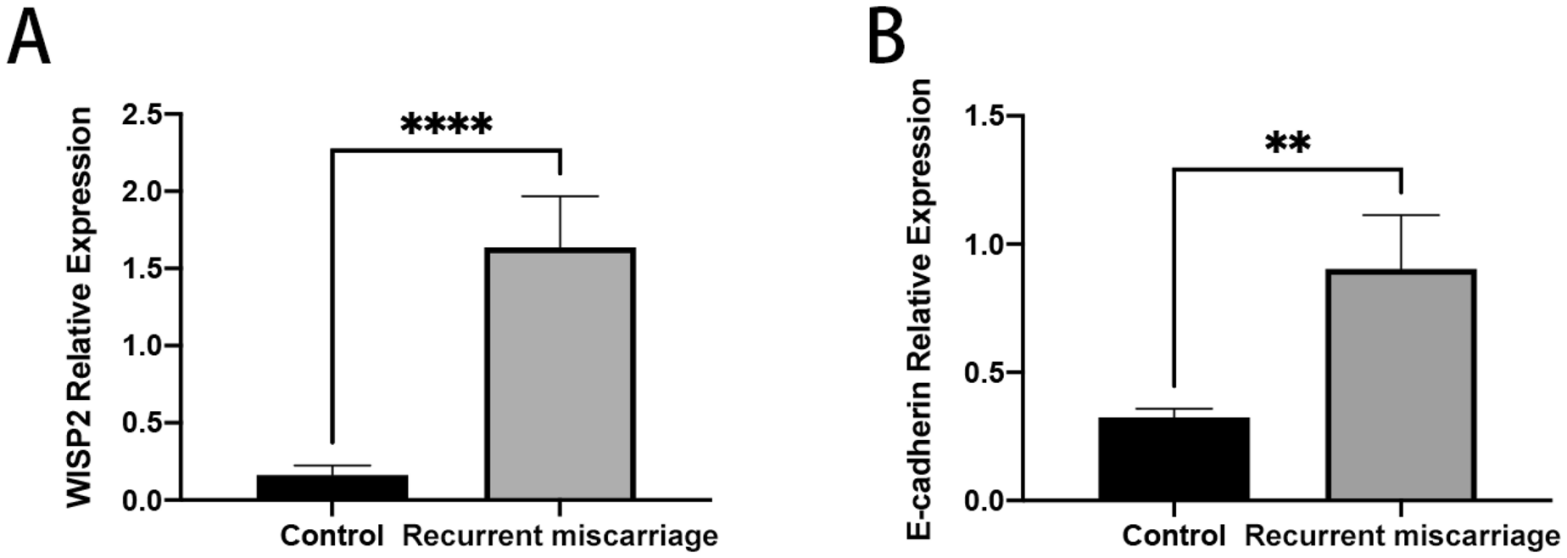
E‐cadherin and WISP2 mRNA expression in the chorion of recurrent miscarriage and normal control was detected using quantitative real‐time PCR. (A) The expression of WISP2 mRNA in the chorion of recurrent miscarriage was significantly higher than that of the normal control. (B) The expression of E‐cadherin mRNA in the chorion of recurrent miscarriage was significantly higher than that of normal control. **p < 0.01, ****p < 0.0001.

### 3.4 The Expression of E‐Cadherin and WISP2 Protein in the Chorion of Recurring Miscarriages and Normal Control Detected by Western Blot

To analyze the protein levels of WISP2 and E‐cadherin in chorionic villi of RM and normal pregnancy controls, chorionic villi proteins were detected by western blotting. The results showed that the expression level of WISP2 protein in the chorionic villi of the missed‐abortion group was statistically significant compared to that in the normal pregnancy group (*p* < 0.01) (Figure 4A,B). The E‐cadherin protein expression level in the chorionic villi of the RM group is significantly up‐regulated in comparison to the normal pregnancy group, highly significantly different from (*p* < 0.0001) (Figure 4A,C). The protein expression levels of WISP2 and E‐cadherin in the chorionic villi of patients with RM exhibited a prominent increase.

**FIGURE 4:**
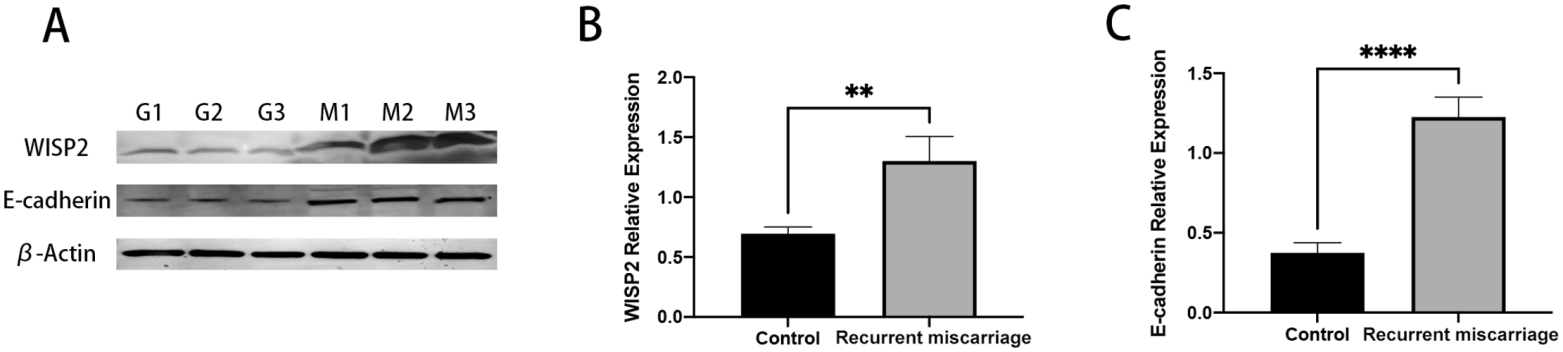
E‐cadherin and WISP2 protein expression in the chorion of recurrent miscarriage and normal control were detected by Western blot. (A) Western blot detected WISP2 and E‐cadherin protein expression in the chorion of recurrent miscarriage and normal control. (B) WISP2 protein expression in the chorion of recurrent miscarriage was significantly higher than that in normal control. (C) E‐cadherin protein expression in the chorion of recurrent miscarriage was significantly higher than that of normal control. **p < 0.01, ****p < 0.0001.

### 3.5 WISP2 and E‐Cadherin Protein Expression in Chorion of Recurring Miscarriages and Normal Control Detected by IHC

Therefore, we still need to explore the localization and expression of WISP2 and E‐cadherin in addition to normal chorion; this study employed immunohistochemical techniques to thoroughly examine protein expression in chorion samples. Notably, the protein expression of WISP2 and E‐cadherin was observed in the chorion of both RM and normal controls. WISP2 positive staining was also observed in the cytoplasm and nucleus of the syncytiotrophoblasts and cytotrophoblasts. Among them, the cytoplasmic positive staining of trophoblasts was the strongest (Figure 5A). Closer analysis revealed that E‐cadherin appeared in both syncytiotrophoblasts and cytotrophoblast cells, and its positivity appeared in the cell membrane, cytoplasm, and nuclear areas. Among these, the positive staining of the cell membrane was the strongest. According to the analysis, for the miscarriage group, the mean optical density (AOD) of WISP2 in the chorion was significantly higher than that of the normal control group (*p* < 0.05) (Figure 5B). Similarly, the average optical density (AOD) of E‐cadherin in the chorion of miscarriage cases showed a significant increase compared to that of the control group (*p* < 0.05) (Figure 5C).

**FIGURE 5:**
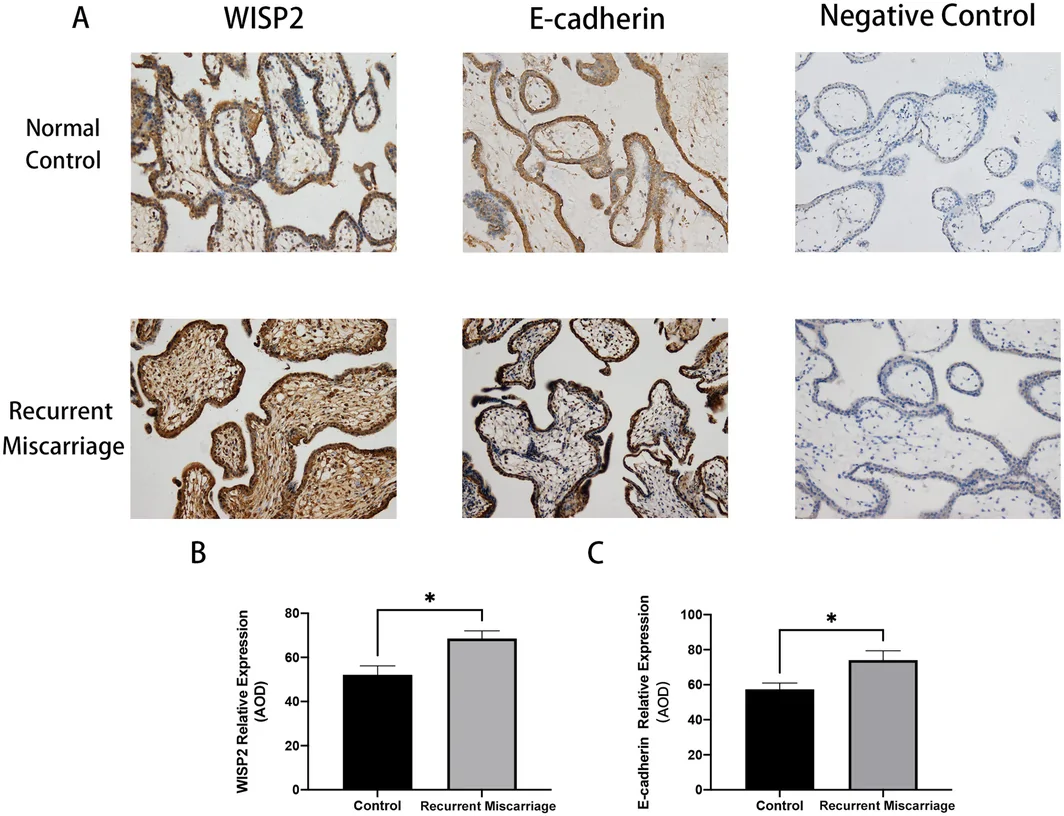
WISP2 and E‐cadherin protein expression in the chorion of recurrent miscarriage and normal control detected by IHC. (A) Immunohistochemical staining of WISP2 and E‐cadherin in the chorion of women with recurrent miscarriage and normal early pregnant women. (B) Results of AOD of WISP2 quantitative analysis. (C) The AOD of E‐cadherin in the chorion of recurrent miscarriage was significantly higher than that of normal control. *p < 0.05.

### 3.6 Overexpression of WISP2 Inhibits Cell Growth

In a study investigating the effect of WISP2 on RM, we validated the overexpression of WISP2 in HTR‐8 cells by introducing a plasmid containing the WISP2 coding sequence using western blotting. The results showed that after transfection with WISP2 cDNA, the level of WISP2 in HTR‐8 cells significantly increased (Figure 6A,B) (*p* < 0.01). At the same time, we assessed the proliferation of transfected cells using the MTT assay. The results indicated that the overexpression of WISP2 led to approximately 35% inhibition of HTR‐8 cell proliferation, which was statistically significant (*p* < 0.0001) (Figure 6C).

**FIGURE 6:**
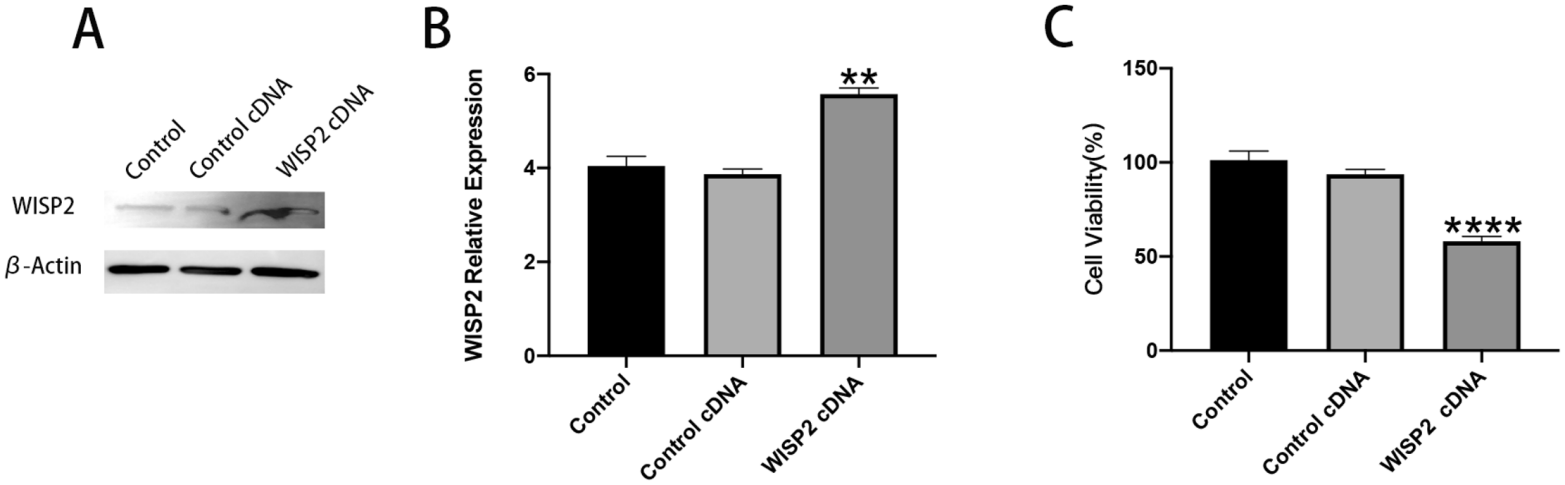
WISP2 overexpression inhibits cell activity. (A) Western blot analysis of WISP2. (B) Quantitative analysis of WISP2 was significantly overexpressed. (C) MTT assay was used to detect the activity of HTR‐8 cells in different groups. ** and **** represent *p* < 0.01, *p* < 0.0001, respectively.

### 3.7 WISP2 Overexpression Retards the Process of Cell Transfer

The function of WISP2 regulates the movement and penetration of HTR‐8 cells was proven. High‐expression experiments of the WISP2 gene were conducted in HTR‐8 cells to assess their migration behavior using a wound healing model. We found that high expression of WISP2 significantly reduced the migratory activity of HTR‐8 cells. After 24 h of healing, there were no statistically significant differences in the cellular motility of the WISP2 cDNA cohort relative to that of the control cDNA cohort (*p* > 0.05), although a decreasing trend was observed. However, after 48 h of healing, the cell migration ability of the WISP2 cDNA group decreased significantly (*p* < 0.0001), with a reduction of approximately 65% compared with the control cDNA group (Figure 7A–C). Furthermore, through Matrigel invasion experiments, our experimental results showed that high WISP2 expression markedly inhibited the invasion ability of cells, with the invasion ability dropping by 40% (*p* < 0.01) compared to the control cDNA group (Figure 7D,E), indicating that WISP2 can inhibit the process of cell transfer.

**FIGURE 7:**
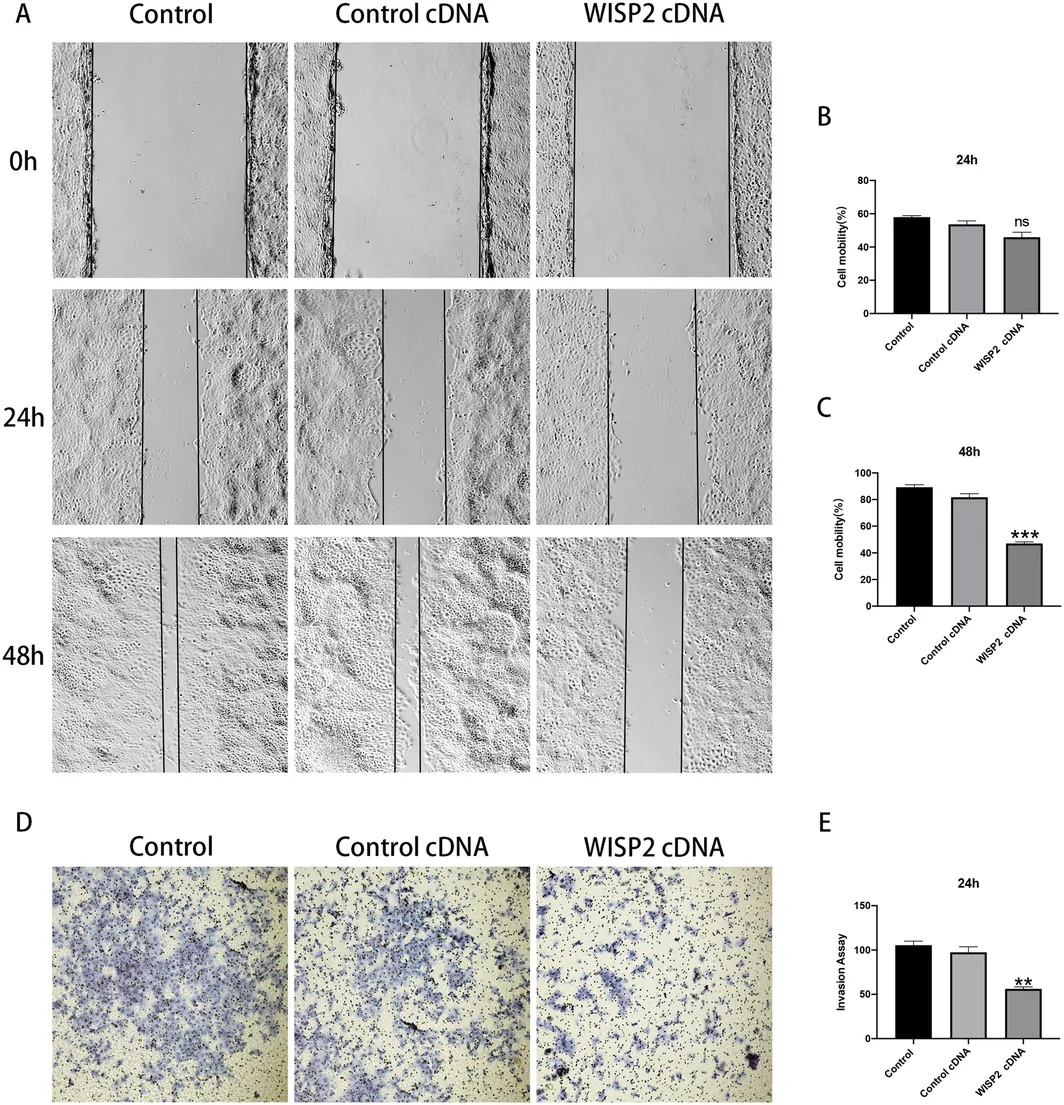
WISP2 overexpression retards cell migration and invasion. (A) A wound‐healing assay was performed to detect the migration of cells in different groups. (B) Compared with the control cDNA group, there was no significant difference in cell mobility at 24 h. (C) The cell mobility at 48 h was significantly lower than that of the control cDNA group. (D) The invasiveness of HTR‐8 cells in different groups was detected by the Transwell invasion assay. (E) The cell invasion at 24 h was significantly lower than that in the control cDNA group. ns represent no significant difference, **p < 0.01, ***p < 0.001.

### 3.8 Overexpression of WISP2 Can Activate ERK and Increase the Expression of E‐Cadherin

In this study, we investigated the role of WISP2 in natural abortion by determining whether the overexpression of WISP2 in HTR‐8 cells might have an impact on ERK, P‐ERK, E‐cadherin and Vimentin protein expression. Western blot analysis after overexpression of WISP2 in HTR‐8 cells revealed a large increase in E‐cadherin protein expression (*p* < 0.05) (Figure 8A,B). Although the study did not show significant changes in the overall expression of ERK1/2 (*p* > 0.05), a notable increase in phosphorylated ERK and a decline in vimentin protein expression were observed (*p* < 0.05) (Figure 8C–E). These observations suggest that WISP2 overexpression promotes an epithelial‐like phenotype in trophoblast cells, accompanied by activation of the ERK signaling pathway.

**FIGURE 8:**
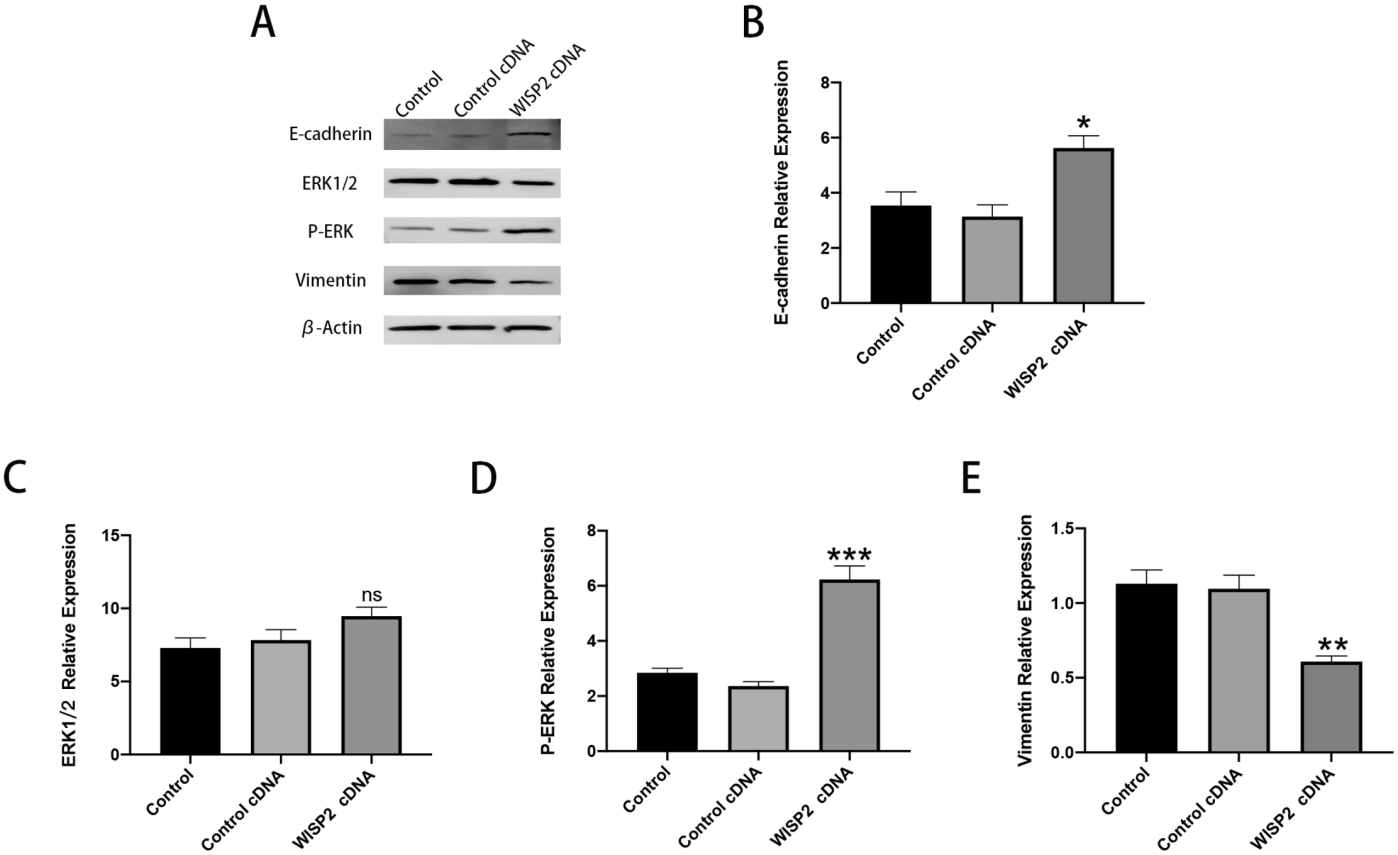
WISP2 overexpression can activate ERK and up‐regulate the expression of E‐cadherin. (A) Western Blotting was used to detect the expression of E‐cadherin, ERK1/2, P‐ERK, and Vimentin in cells of different groups. (B) The expression of E‐cadherin was a quantitative analysis. (C) There was no significant difference in the expression of ERK1/2 between the WISP2 overexpression group and the control cDNA group. (D) P‐ERK expression was a quantitative analysis. (E) The expression of Vimentin was a quantitative analysis. ns represent no significant difference, *, **, and *** represent *p* < 0.05, *p* < 0.01, *p* < 0.001, respectively.

## 4 Discussion

So far, the role of WISP2 in the process of miscarriage is still in the exploratory stage; however, its effectiveness in various types of cancer has been clearly elucidated (Chai et al. 2019; Ferrand et al. 2022; Jia et al. 2021). The involvement of WISP2 in carcinogenesis remains unidentified even if works are pointing out the variation of expression features and of its functions according to the type of the different tumors (Berger et al. 2019). The inconsistency that comes from these differences can only obscure more the concept of involvement of WISP2 in processes related to cell proliferation, migration and invasion (Shi et al. 2020). WISP2 as a promoter in MCF‐7 cells, also augment the proliferation cells in response to epidermal growth factor but, in response to PCa cells they are inhibited in growth (Inadera et al. 2000; Ray et al. 2005). WISP2 is shown to mediate the epithelial‐mesenchymal transition (EMT) process by regulating the expression of metalloproteinases (MMP)9 and MMP‐2 via the ERK signaling pathway, thereby controlling the proliferation, invasion, and metastasis of gastric carcinoma cells (Barreto et al. 2016). Moreover, Shi et al. found that it could decrease P‐ERK expression, so WWISP2 might be able to suppress it (Shi et al. 2020). ERK1/2 signaling pathway was thought to block ovarian cancer cell proliferation and apoptosis. Subsequent research has shown that WISP2 downregulates Snail and MMP‐13 levels in ovarian cancer cells. WISP2 is speculated to regulate the epithelial‐mesenchymal transition (EMT) of ovarian cancer cells, consequently facilitating ovarian cancer cell migration and invasion. E‐cadherin, an important cell invasion and EMT molecule, is overexpressed and cannot be overlooked. Although significant progress has been made in the study of WISP2 in various cancers, its relationship with miscarriage requires further in‐depth research.

In early pregnancy, cytotrophoblasts (CTB) of the inner villi fuse to form syncytiotrophoblasts (STB) (Aplin 2010). The anchored villi attach to the maternal decidua and proliferate, form trophoblast columns, break through syncytiotrophoblasts, and differentiate into extravillous trophoblasts (EVT). Moreover, EVT can invade and remodel the uterine spiral artery and implant in the maternal decidua so that the placenta can obtain adequate blood supply (Red‐Horse et al. 2004). Notably, the stable development of anchored villi is a prerequisite for maintaining the invasiveness of EVT and remodeling the maternal uterine spiral artery (Milovanov et al. 2012). Research shows that in the pregnant uterus, EVT is distributed in at least four distinct regions, including columnar EVT located at the tips of the chorionic cell columns, interstitial EVT in the decidua interacting with maternal leukocytes, intravascular EVT in contact with the maternal vascular endothelium within the uterine spiral arteries, and villous EVT within the chorionic tissue adjacent to the maternal decidua (Brosens et al. 2002). Therefore, each early fetal development process plays an indispensable role at its specific stage, laying the foundation for the healthy growth and maturity of the fetus.

In the early stages of pregnancy, the proliferation and invasive characteristics of trophoblast cells resemble those of cancer cells but are precisely regulated, which is crucial for embryo maturation and the smooth progress of pregnancy. If the invasive ability of trophoblasts is insufficient, the uterine spiral artery will retain its contractile function. Excessive blood flows to the villous space, and oxygen partial pressure in developing embryos and placenta increases (Burton and Jauniaux 2004; Burton et al. 2009). As a result, it leads to a variety of diseases such as recurrent spontaneous abortion, gestational hypertension, fetal intrauterine growth restriction, and so on (Burton and Jauniaux 2011; Gyselaers and Peeters 2013). It is known that epithelial‐to‐mesenchymal transition (EMT) is significant in the process of implantation in embryogenesis and the establishment of the placenta (Vićovac and Aplin 1996). Epithelialcells are stimulated to transform into motile mesenchymal cells, and this transformation is an important contributing factor (Kalluri and Weinberg 2009). During EMT, epithelial cells lose their polarity, reduce their intercellular adhesion, and increase their mobility (Lee et al. 2006). Importantly, it allows trophoblast cell columns to invade decidual stroma in the first and second trimester of pregnancy (Kokkinos et al. 2010). Once the implantation process is over, the EMT process of the cell stops, and the EVT returns to the epithelial phenotype (Floridon et al. 2000).

Therefore, we examined the expression of both WISP2 and E‐cadherin in term placental samples that were not aborted quickly. The expression of both WISP2 and E‐cadherin increased, as indicated by the results, suggesting that chorions that were not timely terminated may exhibit less invasive epithelial morphology. We used the HTR‐8/SVneo cell line as an early embryonic EVT invasion and migration model, constructed WISP2 overexpression cells to simulate EVT of RM, and studied the mechanism of WISP2 in EVT. Research has shown that high levels of WISP2 expression can effectively inhibit the proliferation, metastasis, and invasion abilities of EVT. Mechanistically, the high expression of WISP2 activates the ERK1/2 pathway in EVT, and vimentin expression is downregulated, while the expression of E‐cadherin is increased, which together promote EVT to restore the epithelial cell characteristics.

Nevertheless, several limitations of this study should be acknowledged. First, the relatively small sample size may limit the statistical power and generalizability of our findings; therefore, larger independent cohorts are warranted to validate our conclusions. Second, our functional investigations were confined to in vitro cellular models. While the HTR‐8/SVneo cell line is a well‐established surrogate for human extravillous trophoblasts, future studies employing trophoblast‐specific conditional knockout or overexpression mouse models, or humanized placental xenograft systems, will be essential to further consolidate the in vivo pathophysiological role of WISP2 in miscarriage. Third, although E‐cadherin was selected as a representative marker to evaluate EMT status in clinical tissues, we acknowledge that a more comprehensive panel of EMT‐related regulators, including N‐cadherin, Vimentin, Snail, and ERK pathway components, would provide a more complete mechanistic picture. Future investigations with expanded marker panels are needed to fully delineate the molecular pathways through which WISP2 modulates trophoblast invasion.

## 5 Conclusion

The expression of WISP2 and E‐cadherin markedly increases in the placental villous tissue during miscarriage, a process mainly dependent on the role of WISP2. WISP2 overexpression can inhibit trophoblast cell growth, migration, and invasion; thus, P‐ERK, vimentin, and E‐cadherin levels in trophoblast cells are controlled by WISP2, which prevents EMT. It has been suggested that the overexpression of WISP2 may reduce the invasion ability of extravillous trophoblasts to the maternal decidua, leading to insufficient spiral artery remodeling and adverse pregnancy outcomes. Our findings suggest that restraining WISP2 overabundance can prove to be a successful approach for the prevention and intervention of RM.

## Author Contributions

**Zhe Leng:** conceptualization, data curation, investigation, formal analysis, writing – original draft. **Boyu Yan:** formal analysis, supervision, validation, visualization, writing – original draft. **Fang Wang:** data curation, investigation, project administration, software, writing – original draft. **Xinyi Bian:** conceptualization, formal analysis, validation, funding acquisition, writing – review and editing. **Yu Huang:** methodology, project administration, software, resources, writing – review and editing. **Hongjuan Lv:** conceptualization, data curation, formal analysis, investigation, funding acquisition, writing – original draft. **Zhou Jiang:** conceptualization, data curation, formal analysis, investigation, writing – original draft. **Xianli Sun:** conceptualization, visualization, supervision, writing – review and editing.

## Funding

This work was supported by Medical and Health Scientific Research Project of Qingdao (2026XKRC‐002) and Natural Science Foundation of Shandong Province (ZR2024QH397).

## Ethics Statement

Ethical approval for this study was granted by the Ethics Committee of the Women and Children's Hospital of Qingdao University (no. QFELL‐YJ‐2020‐58), in accordance with the Declaration of Helsinki.

## Consent

Written informed consent was obtained from each participating patient.

## Conflicts of Interest

The authors declare no conflicts of interest.

## Supporting information


**Figure S1:** The copy number of WISP2 in normal and recurrent miscarriage was compared based on three different databases. (A) GSE161969. (B) GSE111974. (C) GSE165004. * and **** represent *p* < 0.05, *p* < 0.0001, respectively.
**Table S1:** Characteristics of the recurrent miscarriage and control.

## Data Availability

The data that support the findings of this study are available on request from the corresponding author. The data are not publicly available due to privacy or ethical restrictions.
