# Supplementary material for: Revealing the Action Mechanism of WISP2 in Miscarriage Based Multi‐Omic Analysis and Experimental Validation
Source: Chem Biol Drug Des. 2026 Nov 5;108(5):e70423. doi: 10.1111/cbdd.70423 (PMC13740338; doi:10.1111/cbdd.70423)
Supplement: Supplementary file 1 [file CBDD-108-e70423-s001.docx]

**Supplementary Material**

**Table S1.** Characteristics of the Recurrent miscarriage and Control^a^.

| Characteristics | Control | Recurrent miscarriage | *P* value |
| --- | --- | --- | --- |
| N | 20 | 20 |  |
| Maternal age, years | 26.50±0.6863 | 27.60±0.6300 | 0.2450 |
| Gestation age, days | 53.35±1.094 | 55.45±0.9360 | 0.1528 |
| Gravidity, orders | 1.600±0.3728 | 1.450±0.2233 | 0.7318 |

^a^Data are presented as mean±SEM. No statistically significant differences in maternal age, gestation age, and gravidity were identified between recurrent miscarriage patients and controls.

Fig. S1 The copy number of WISP2 in normal and recurrent miscarriage was compared based on three different databases. (A) GSE161969. (B) GSE111974. (C) GSE165004. * and **** represent *P* < 0.05, *P* < 0.0001, respectively.
